# The effect of premorbid features on post-stroke rehabilitation outcome

**Published:** 2018-01-05

**Authors:** Ozgur Zeliha Karaahmet, Ebru Umay, Eda Gurcay, Azize Serçe, Ibrahim Gundogdu, Aytul Cakci

**Affiliations:** Department of Physical Medicine and Rehabilitation, Diskapi Yildirim Beyazit Education and Research Hospital, Ankara, Turkey

**Keywords:** Stroke, Rehabilitation, Cerebral Infarction, Prognosis, Rehabilitation Outcome

## Abstract

**Background:** A wide variety of factors influence stroke prognosis, including age, stroke severity, stroke mechanism, infarct location, comorbid conditions, clinical findings, and related complications. The aim of this study was to detect the prognostic determinants in patients with acute stroke for guiding rehabilitation.

**Methods:** Patients with ischemic acute stroke were included in the study. Patients’ age, sex, education level, and marital status, premorbid personality traits, comorbidities such as current smoking status and alcohol consumption, regular exercise habits, and sleeping disorder were recorded. Motor assessment and daily activity skills were evaluated according to the Brunnstrom staging and Functional Independence Measure (FIM), respectively.

**Results:** A total of 85 patients were studied. All patients’ motor and functional stages were significantly improved with the rehabilitation. The improvements in the upper extremity motor levels were less in whom over 76 years and smokers, in patients who had 4 and more comorbidities and sleep disorders. The functional improvement was less in whom over 76 years and men, and in patients who had 4 and more comorbidities and sleep disorders.

**Conclusion:** The significant post-stroke predictor of insufficiency in functioning was having 4 or more risk factors.

## Introduction

In 2010, stroke was reported as the second most common cause of death and the third most common cause of reduced disability-adjusted life-years worldwide.^1^ Rates of patients with post-stroke disability are changing worldwide, but different studies have recorded that stroke prognosis is related with patient age, stroke type, seriousness, place and length of stroke, and family history.^2^

Numerous factors affect rehabilitation outcome in patients with stroke. Several studies have been made in this regard and there will be further ones. So, why is it so important to know the prognostic factors? There are valid reasons for that, notably, clinicians are often asked to predict outcome after stroke by the patient, family, other healthcare workers, and insurance providers. Accurate prognostic models for patients with subacute stroke would have several important uses, such as guiding patient management (allowing more reliable information to be given to patients and their relatives), and improving the planning of patient rehabilitation and discharge.^3^ Forecasts of recovery from stroke have focused on the risk factors after the disease developed.

Unfortunately, there are no models for examining the impact of the patient's premorbid condition directly. In this study, we aimed to evaluate the effect of rehabilitation outcome of premorbid features in the patients treated in our clinic due to stroke rehabilitation.

## Materials and Methods

This cross-sectional study was performed prospectively. Patients with stroke attending the Physical Medicine and Rehabilitation Clinic, Ankara, Turkey, were enrolled this study. Study participation was totally voluntary, and patients were informed about the nature of the study. All procedures were in consistency with the Helsinki Declarations of 1975. The study was confirmed by the local institutional ethical committee.

In this study, patients aged between 18-80 years who had first stroke with middle cerebral artery (MCA) ischemic lesion, were in the first 7-14 days after stroke, and were hospitalized and rehabilitated for 1 month were included. Unconscious patients and patients with limited cooperation, sensory aphasia, premorbid antidepressant use, recurrent stroke, or bilateral hemiplegia were excluded from the study.

Patients’ age, sex, education level and marital status, premorbid personality traits (defined as self-reported history of peacefull, aggresive, withdrawn, and anxious) were recorded. Subjects were interviewed using the Structured Clinical Interview for Diagnostic and Statistical Manual (DSM) of personality disorders (SCID-II).^4^

Additionally, patients were evaluated for the presence of comorbidities [hypertension (HT), diabetes mellitus (DM), heart disease, asthma, hyperlipidemia, and gout], current smoking status and alcohol consumption, regular exercise habits (defined as more than 30 minutes daily walk), and sleeping disorder.^5^^,^^6^

The condition of sleep was questioned by asking about the following: difficulty falling asleep, taking or being dependent on medication to help one sleep, sleep interrupted during the night, difficulty sleeping (falling/staying asleep) owing to moods or tension, difficulty sleeping owing to pain or itching, inability to return to sleep after waking at night, waking early or feeling tired, and sleeping more than two hours during the day.^6^

Patients were divided into 3 groups for age (between 40-65, 66-75, and 76 years and older), also for body mass index (underweight ≤ 18.5 kg/m^2^, normal weight = 18.5-24.9 kg/m^2^, and overweight ≥ 25 kg/m^2^).

The motor assessment was done according to the Brunnstrom staging which evaluates and interprets motion patterns according to the stage of motor function recovery.^7^ Daily activity skills were evaluated according to the Functional Independence Measure (FIM) at the time of hospitalization and discharge. The FIM scale defines physical and cognitive disability. This scale points on the load of care. Items are put on the level of aid recurred for personal to handle activities of daily living (ADL). The measure contains 18 items. Every item is counted from 1 to 7 based on the grade of independence, where 1 indicates total dependence and 7 indicates complete independence. The scores change from 18 to 126.^8^

The combination of the significant risk factors was established. Risk combination groups were compared with FIM which was applied pre-treatment, after-treatment, and in terms of changing.

Whole patients attended in the rehabilitation plan for five days a week for a month. Traditional and neurophysiological therapy techniques were accessed.

Data analyses were made using SPSS for Windows software (version 15, SPSS Inc., Chicago, IL, USA). The continuous variables were evaluated with the Kolmogorov-Smirnov test as to whether or not they were different from the normal distribution. Descriptive statistics were shown as a mean ± standard deviation (SD) for continuous variables and frequencies and percentages (%) for nominal variables using chi-square test. Statistically, significant differences in repeated measurements within the group were evaluated with the Wilcoxon Signed Rank test. The variables were compared between two groups using Man-Whitney U test and 3 or more properties were evaluated by Kruskal-Wallis test. Subgroup analyses were performed for the results which were meaningful. Values of P < 0.050 were considered statistically significant.

## Results

A total of 102 patients were included, but the study was completed with 85 patients due to problems such as recurrent stroke, acute myocard infarction, or the patients own request to be discharged less than one month. 

**Table 1:** Patients' premorbid characteristics and the distribution and comparison of pre- and posttreatment evaluation parameters (n = 85)

**Variable**	**Value**
Age (year) (mean ± SD)	68.69 ± 11.07
Gender [n (%)]	
Women	36 (42.4)
Men	49 (57.6)
Educational status [n (%)]	
Illiterate	14 (16.5)
Under 5-year	9 (10.6)
5-year	50 (58.8)
More than 5 years	12 (14.1)
Marital status [n (%)]	
Married	61 (71.8)
Single	0 (0)
Divorced/widow	24 (28.2)
Comorbidities [n (%)]	
Presence of comorbidities	70 (82.4)
1 comorbidity	25 (35.7)
2 comorbidities	25 (35.7)
3 comorbidities	14 (20.0)
≥ 4 comorbities	6 (8.6)
Number of smoker [n (%)]	29 (34.1)
Number of patient using alcohol [n (%)]	0 (0.0)
Number of patint with sleep disorder [n (%)]	61 (71.8)
Number of unbalanced or undernourished patient [n (%)]	
Underweight	16 (18.8)
Normal	56 (65.9)
Overweight	13 (15.3)
Patient with lack of exercise [n (%)]	77 (90.6)
Premorbid personality [n (%)]	
Peacefull	35 (41.2)
Aggresive/short fuse	23 (27.1)
Withdrawn	16 (18.8)
Anxious	11 (12.9)
BT Brunstroom stage (1-6) (mean ± SD)	
Upper extremity	3.16 ± 1.57*
Hand	3.01 ± 1.56*
Lower extremity	3.65 ± 1.07*
AT Brunstroom stage (1-6) (mean ± SD)	
Upper extremity	4.04 ± 0.92
Hand	4.10 ± 0.84
Lower extremity	4.42 ± 0.56
BT functional questionnaire score (0-14) (mean ± SD)	7.29 ± 3.10*
AT functional questionnaire score (0-14) (mean ± SD)	2.54 ± 2.88
BT FIM total score (18-126) (mean ± SD)	71.28 ± 33.99*
AT FIM total score (18-126) (mean ± SD)	110.44 ± 20.95

SD: Standard deviation; FIM: Functional independence measure; BT: Before treatment; AT: After treatment

Wilcoxon Signed Rank test was used for the comparison of before- and after-treatment data.

* Significant difference (P < 0.050)

Patients' demographic data, premorbid characteristics, and the distribution of pre- and post-treatment evaluation parameters are shown in table 1. 

The hand, and upper and lower extremity motor and functional stages assessed by Brunnstrom and FIM were significantly improved with the rehabilitation (P = 0.001 for all). According to patients' demographic data and premorbid characteristics, the distributions and comparisons of the Brunnstrom and FIM scores of before and after the treatment and the changes in terms of these scores are shown in tables 2-5.

**Table 2:** The distribution and comparison of pre- and posttreatment Brunstroom stages according to premorbid features

**Brunstroom stage**	**Upper extremity**		**Hand**		**Lower extremity**	
**Variable**	**BT**	**AT**	**BT**	**AT**	**BT**	**AT**
	**Mean ± SD**	**Mean ± SD**	**Mean ± SD**	**Mean ± SD**	**Mean ± SD**	**Mean ± SD**
Age (year)**						
40-65 (n = 28, 32.9%)	3.42 ± 1.81*	4.17 ± 1.05*	3.42 ± 1.81*	4.28 ± 0.89*	3.82 ± 1.33*	4.46 ± 0.63*
66-75 (n = 27, 31.8%)	3.29 ± 1.38*	4.11 ± 0.89*	3.18 ± 1.27*	4.18 ± 0.78*	3.66 ± 1.03*	4.33 ± 0.55*
≥76 (n = 30, 35.3%)	2.40 ± 1.49*	3.16 ± 0.81*	2.16 ± 1.43*	3.06 ± 0.81*	2.50 ± 0.82*	3.16 ± 0.50*
Gender†						
Women	2.94 ± 1.47	3.94 ± 0.95	2.80 ± 1.52	3.97 ± 0.81	3.33 ± 0.92	4.30 ± 0.46
Men	3.32 ± 1.65	4.12 ± 0.90	3.16 ± 1.58	4.20 ± 0.86	3.89 ± 1.12	4.51 ± 0.61
Educational status**						
Illiterate (n = 14, 16.5%)	2.92 ± 1.32	3.92 ± 0.82	2.57 ± 1.45	3.71 ± 0.72	3.35 ± 0.84	4.28 ± 0.46
Under 5 years (n = 9, 10.6%)	2.67 ± 1.01	3.88 ± 0.78	2.66 ± 1.09	4.22 ± 0.67	3.34 ± 0.51	4.34 ± 0.52
5 years (n = 50, 58.8%)	3.36 ± 1.71	4.12 ± 0.96	3.20 ± 1.65	4.18 ± 0.89	3.80 ± 1.14	4.51 ± 0.58
More than 5 years (n = 12, 14.1%)	3.02 ± 1.65	4.02 ± 1.04	3.02 ± 1.65	4.16 ± 0.83	3.67 ± 1.30	4.34 ± 0.65
Marital status†						
Married (n = 61, 71.8%)	3.03 ± 1.59	3.96 ± 0.94	2.91 ± 1.51	4.04 ± 0.84	3.62 ± 1.11	4.40 ± 0.58
Divorced/widow (n = 24, 28.2%)	3.50 ± 1.53	4.25 ± 0.84	3.25 ± 1.67	4.25 ± 0.84	3.75 ± 0.98	4.45 ± 0.51
Comorbidities**						
1 comorbidity	3.04 ± 1.67*	3.96 ± 1.01*	2.84 ± 1.59*	4.08 ± 0.86*	3.60 ± 1.08*	4.52 ± 0.51*
2 comorbidities	3.05 ± 1.42*	4.01 ± 0.91*	2.96 ± 1.39*	4.16 ± 0.80*	3.44 ± 0.91*	4.24 ± 0.52*
3 comorbidities	3.64 ± 1.59	4.28 ± 0.82	3.64 ± 1.59	4.28 ± 0.82	4.07 ± 0.91	4.57 ± 0.51
≥ 4 comorbities	4.51 ± 0.54*	4.67 ± 0.51*	4.51 ± 0.54*	4.67 ± 0.51*	4.50 ± 0.54*	4.83 ± 0.41*
Smoking†						
Yes (n = 29, 34.1%)	2.14 ± 1.37*	3.22 ± 0.77*	2.14 ± 1.37*	3.16 ± 0.83*	3.41 ± 1.18	4.27 ± 0.64
No (n = 56, 65.9%)	3.53 ± 1.56*	4.26 ± 0.92*	3.30 ± 1.58*	4.23 ± 0.83*	3.78 ± 1.01	4.50 ± 0.51
Sleep†						
Normal (n = 24, 28.3%)	3.45 ± 1.53*	4.54 ± 0.86*	3.29 ± 1.49*	4.29 ± 0.80*	3.38 ± 1.08*	4.52 ± 0.59*
Disturbed (n = 61, 71.8%)	2.26 ± 1.23*	3.04 ± 0.88*	2.04 ± 1.30*	3.02 ± 0.76*	2.23 ± 0.82*	3.26 ± 0.58*
BMI**						
Normal (n = 56, 65.9%)	3.46 ± 1.62*	4.53 ± 0.92*	3.21 ± 1.57*	4.19 ± 0.84*	3.24 ± 1.15*	4.48 ± 0.58*
Underweight (n = 16, 18.8%)	2.56 ± 1.31	3.75 ± 0.93	2.77 ± 1.45	3.75 ± 0.86	3.05 ± 0.68	4.08 ± 0.40
Overweight (n = 13, 15.3%)	2.17 ± 0.87*	3.37 ± 0.57*	2.14 ± 1.17*	3.04 ± 0.43*	2.12 ± 0.92*	3.03 ± 0.57*
Exercise†						
Insufficient (n = 77, 90.6%)	2.12 ± 1.55	3.62 ± 0.91	2.12 ± 1.55	3.62 ± 0.91	3.12 ± 1.55	4.37 ± 0.51
Regular (n = 8, 9.4%)	3.27 ± 1.55	4.09 ± 0.92	3.10 ± 1.54	4.15 ± 0.82	3.71 ± 1.01	4.42 ± 0.57
Premorbid personality**						
Peacefull (n = 35, 41.2%)	3.31 ± 1.73*	4.13 ± 1.02*	3.26 ± 1.73*	4.15 ± 0.91*	3.77 ± 1.11*	4.52 ± 0.54*
Aggresive (n = 23, 27.1%)	3.16 ± 1.35*	4.10 ± 0.70*	3.21 ± 1.34*	4.13 ± 0.76*	3.69 ± 0.98*	4.36 ± 0.59*
Withdrawn (n = 16, 18.8%)	3.01 ± 0.18	4.03 ±0.82	3.01 ± 0.18	4.01 ± 0.54	3.05 ± 0.27	4.01 ± 0.13
Anxious (n = 11, 12.9%)	2.02 ± 0.32*	3.04 ± 0.19*	2.02 ± 0.32*	3.04 ± 0.99*	2.15 ± 0.57*	3.21 ± 0.19*

SD: Standard deviation; BT: Before treatment; AT: After treatment; BMI: Body mass index

* Significant difference (P < 0.050),

** Kruskal-Wallis test,

† Man-Whitney U test

The improvements in the upper extremity motor functional stages assessed by Brunnstrom were less in whom over 76 years and smokers, and in patients who had 4 and more comorbidities and sleep disorders. Similar results were seen in Brunnstrom hand motor levels except for the age. The only parameter that affected the lower extremity Brunnstrom level was sleeping disorder. The functional improvement evaluated by FIM was less in whom over 76 years and men, and in patients who had 4 and more comorbidities and sleep disorders. 

According to the significant risk combination groups (over 76 years, male gender, 4 and more comorbidities, smoking, sleep disorders, obesity, and anxious personality trait) which were found in pretreatment, posttreatment and the changes in terms of total FIM scores, the distribution and comparison of the FIM levels are shown in table 6.

In risk combination groups, pretreatment and posttreatment FIM levels were significantly lower in whom had 4 and more risk factors than those with 2 and more, also 3 and more risk factors (for 2 and more, P = 0.001, P = 0.001, and for 3 and more, P = 0.001, P = 0.001, respectively).

## Discussion

In recent years, many epidemiological studies have given new insights into old and new lifestyle factors (nutrition, alcohol, tobacco, and education) that influence the risk of cerebrovascular events.^9^ In addition, numerous studies have been made on prognostic factors in patients with acute stroke, while studies on premorbid features as prognostic determinants affecting the post-stroke rehabilitation outcomes are less. In this study, we assessed the effect of patients’ premorbid features on the post-stroke rehabilitation outcomes, and found that advanced age, the excess number of comorbidities, smoking, sleep disorders, obesity, and anxious personality were negative prognostic factors.

**Table 3:** The comparison and distribution of pre- and posttreatment Functional Questionnaire and functional independence measure (FIM) score according to premorbid features

**Score**	**Functional questionnaire**		**FIM**	
**Variable**	**BT**	**AT**	**BT**	**AT**
Age (year)**	Mean ± SD	Mean ± SD	Mean ± SD	Mean ± SD
40-65 (n = 28, 32.9%)	6.32 ± 3.24*	2.39 ± 2.67*	67.13 ± 27.44*	105.40 ± 21.71
66-75 (n = 27, 31.8%)	6.63 ± 2.83*	2.44 ± 3.21*	74.59 ± 41.25	113.20 ± 14.98
≥ 76 (n = 30, 35.3%)	7.92 ± 3.09*	3.76 ± 2.81*	80.03 ± 30.30*	111.25 ± 15.50
Gender†				
Women	8.36 ± 2.35*	2.97 ± 3.01	54.38 ± 29.23*	108.75 ± 14.69
Men	6.51 ± 3.36*	2.22 ± 2.77	83.69 ± 32.05*	111.69 ± 17.87
Educational status**				
Illiterate (n = 14, 16.5%)	7.21 ± 2.08	2.14 ± 2.74	67.35 ± 33.37	110.07 ± 13.21
Under 5 years (n = 9, 10.6%)	7.23 ± 1.32	2.32 ± 1.93	68.67 ± 20.01	109.24 ± 12.30
5 years (n = 50, 58.8%)	7.82 ± 3.39	2.54 ± 3.17	72.89 ± 34.98	108.25 ± 16.26
More than 5 years (n = 12, 14.1%)	6.97 ± 3.11	2.65 ± 2.49	71.01 ± 25.21	118.67 ± 08.55
Marital status†				
Married (n = 61, 71.8%)	7.25 ± 3.19	2.71 ± 2.94	72.50 ± 35.09	108.80 ± 11.69
Divorced/widow (n = 24, 28.2%)	6.62 ±2.59	1.89 ± 2.64	68.62 ± 30.99	114.62 ± 11.71
Comorbidities**				
1 comorbidity	8.96 ± 2.79*	2.32 ± 2.44*	81.01 ± 30.67*	119.01 ± 6.09*
2 comorbidities	6.85 ± 2.65*	2.56 ± 2.61*	75.24 ± 23.14	104.72 ± 14.00
3 comorbidities	6.36 ± 2.53	2.71 ± 3.47	74.21 ± 40.92	111.00 ± 15.76
≥ 4 comorbities	4.50 ± 1.64*	2.86 ± 2.93*	63.72 ± 32.31*	98.76 ± 13.29*
Smoking†				
Yes (n = 29, 34.1%)	6.80 ± 2.86	2.10 ± 2.81	78.27 ± 34.32	119.23 ± 05.32
No (n = 56, 65.9%)	7.24 ± 3.36	3.08 ± 3.11	67.73 ± 34.02	107.20 ± 10.12
Sleep†				
Normal (n = 24, 28.3%)	6.72 ± 3.34*	1.83 ± 2.62*	80.37 ± 33.58*	119.54 ± 06.69*
Disturbed (n = 61, 71.8%)	8.75 ± 1.67*	5.33 ± 2.76*	48.16 ± 28.39*	64.95 ± 14.74*
BMI**				
Normal (n = 56, 65.9%)	6.84 ± 3.29*	2.95 ± 2.56*	78.09 ± 33.34*	115.12 ± 09.11*
Underweight (n = 16, 18.8%)	7.29 ± 3.10	3.20 ± 3.34	65.03 ± 13.47	96.86 ± 15.41
Overweight (n = 13, 15.3%)	8.81 ± 1.72*	4.03 ± 2.56*	62.50 ± 23.73*	89.37 ± 29.16*
Exercise†				
Insufficient (n = 77, 90.6%)	7.50 ± 4.62	2.87 ± 2.58	73.12 ± 34.34	106.62 ± 14.37
Regular (n = 8, 9.4%)	7.27 ± 2.94	2.50 ± 2.92	71.09 ± 34.17	110.84 ± 10.70
Premorbid personality**				
Peacefull (n = 35, 41.2%)	6.80 ± 3.03*	2.27 ± 2.91*	72.05 ± 33.36*	117.12 ± 08.81*
Aggresive (n = 23, 27.1%)	7. 45 ± 2.18*	2.64 ± 2.55*	67.63 ± 32.70	112.94 ± 10.83
Withdrawn (n = 16, 18.8%)	7.78 ± 2.96*	3.36 ± 3.57*	68.03 ± 20.41	110.86 ± 13.26
Anxious (n = 11, 12.9%)	9.13 ± 3.42*	5.42 ± 5.81*	58.06 ± 20.28*	94.03 ± 10.92*

SD: Standard deviation; BT: Before treatment; AT: After treatment; FIM: Functional independence measure; BMI: Body mass index

* Significant difference (P < 0.050),

** Kruskal-Wallis test,

† Man-Whitney U test

**Table 4:** The comparison and distribution of treatment effects according to premorbid features based on Brunstroom stage

**Brunstroom stage**	**Upper ** **extremity**	**P** †† **(AT-BT)**	**Hand**	**P** †† **(AT-BT)**	**Lower ** **extremity**	**P** †† **(AT-BT)**
**Variable**	**Mean ± SD**		**Mean ± SD**		**Mean ± SD**	
Age (year)**						
40-65 (n = 28, 32.9%)	0.75 ± 0.88*	0.047	0.85 ± 0.97	0.617	0.64 ± 0.86	0.982
66-75 (n = 27, 31.8%)	0.81 ± 0.78*		1.00 ± 0.83		0.66 ± 0.78	
76 (n = 30, 35.3%)	0.36 ± 0.46*		0.90 ± 0.77		0.66 ± 0.37	
Gender†						
Women	1.00 ± 0.79	0.731	1.16 ± 0.91	0.615	0.97 ± 0.77	0.578
Men	0.79 ± 0.88		1.04 ± 0.86		0.61 ± 0.75	
Educational status**						
Illiterate (n = 14, 16.5%)	1.00 ± 0.78	0.117	1.14 ± 0.86	0.311	0.92 ± 0.73	0.627
Under 5 years (n = 9, 10.6%)	1.22 ± 0.67		1.55 ± 0.88		1.00 ± 0.70	
5 years (n = 50, 58.8%)	0.76 ± 0.79		0.98 ± 0.86		0.70 ± 0.78	
More than 5 years (n = 12, 14.1%)	1.00 ± 0.85		1.16 ± 0.97		0.66 ± 0.88	
Marital status†						
Married (n = 61, 71.8%)	0.93 ± 0.85	0.855	1.13 ± 0.86	0.438	0.78 ± 0.79	0.941
Divorced/widow (n = 24, 28.2%)	0.75 ± 0.84		1.00 ± 0.93		0.70 ± 0.75	
Comorbidities**						
1 comorbidity	0.92 ± 0.86*	0.048	1.24 ± 0.87*	0.004	0.92 ± 0.81	0.053
2 comorbidities	0.96 ± 0.78*		1.20 ± 0.86*		0.80 ± 0.81	
3 comorbidities	0.64 ± 0.92*		0.64 ± 0.84*		0.50 ± 0.65	
≥ 4 comorbities	0.16 ± 0.40*		0.10 ± 0.15*		0.33 ± 0.51	
Smoking†						
Yes (n = 29, 34.1%)	1.47 ± 0.84*	0.026	1.46 ± 0.82*	0.044	0.86 ± 0.83	0.917
No (n = 56, 65.9%)	0.33 ± 0.82*		0.52 ± 0.87*		0.71 ± 0.75	
Sleep†						
Normal (n = 24, 28.3%)	1.37 ± 0.71*	0.032	1.58 ± 0.65*	0.035	1.38 ± 0.77*	0.021
Disturbed (n = 61, 71.8%)	0.68 ± 0.82*		0.70 ± 0.88*		0.43 ± 0.75*	
BMI**						
Normal (n = 56, 65.9%)	1.76 ± 0.57	0.072	2.01 ± 0.05	0.165	1.24 ± 0.80	0.521
Underweight (n = 16, 18.8%)	1.18 ± 0.75		1.37 ± 0.71		0.98 ± 0.57	
Overweight (n = 13, 15.3%)	0.67 ± 0.85		0.98 ± 0.90		0.93 ± 0.68	
Exercise†						
Insufficient (n = 77, 90.6%)	1.50 ± 0.75	0.433	1.50 ± 0.75	0.217	1.25 ± 1.03	0.185
Regular (n = 8, 9.4%)	1.11 ± 0.83		1.20 ± 0.88		0.91 ± 0.74	
Premorbid personality**						
Peacefull (n = 35, 41.2%)	0.81 ± 0.86	0.618	0.89 ± 0.91	0.776	0.75 ± 0.78	0.478
Aggresive (n = 23, 27.1%)	0.83 ± 0.81		0.82 ± 0.87		0.76 ± 0.75	
Withdrawn (n = 16, 18.8%)	1.00 ± 0.70		1.00 ± 0.32		1.00 ± 0.02	
Anxious (n = 11, 12.9%)	1.03 ± 0.05		1.01 ± 0.11		1.08 ± 0.11	

SD: Standard deviation; BT: Before treatment; AT: After treatment; BMI: Body mass index

* Significant difference (P < 0.050),

** Kruskal-Wallis test,

† Man-Whitney U test,

†† Wilcoxon signed rank test

Former informations have tried to detect factors related with stroke prognosis, but results are often contradictory.^10^ Despite the common belief, Formisano, et al. found that the age was not effective on motor recovery.^11^ In a recent study, it was detected that the functional outcome was poor in older patients.^12^ In this study, we divided the patients into 3 groups according to age (40-65, 66-75, and 76 years and over). The functional and upper extremity motor improvements were less in over 76 years. Prior works, based on that it can be caused by the decrease of muscle strength, in older patients was heavier than in younger patients.^10^^-^^12^

Women's lifelong stroke prevalence is lower. But, female gender has been reported as a negative prognostic indicator for the stroke rehabilitation outcome.^13^^,^^14^ Zhang, et al. have found that women are more opportune to cerebral infarction with cardiogenic origin in younger age, also their prognosis are worse.^14^

**Table 5:** The comparison and distribution of treatment effects according to premorbid features based on functional independence measure (FIM) score

**Variable**	**FIM score**	**P** †† ** (AT-BT)**
Age (year)**	Mean ± SD	
40-65 (n = 28, 32.9%)	47.26 ± 8.74*	0.032
66-75 (n = 27, 31.8%)	38.40 ± 32.47*	
76 (n = 30, 35.3%)	31.21 ± 22.06*	
Gender†		
Women	54.36 ± 23.67*	0.001
Men	28.01 ± 20.65*	
Educational status**		
Illiterate (n = 14, 16.5%)	45.71 ± 21.42	0.821
Under 5 years (n = 9, 10.6%)	40.04 ± 24.58	
5 years (n = 50, 58.8%)	43.36 ± 22.19	
More than 5 years (n = 12, 14.1%)	39.25 ± 19.17	
Marital status†		0.362
Married (n = 61, 71.8%)	39.29 ± 26.15	
Divorced/widow (n = 24, 28.2%)	47.01 ± 21.07	
Comorbidities**		
1 comorbidity	45.48 ± 27.36*	0.013
2 comorbidities	42.00 ± 29.57*	
3 comorbidities	37.04 ± 22.96*	
≥ 4 comorbities	29.78 ±21.35*	
Smoking†		
Yes (n = 29, 34.1%)	42.50 ± 24.64	0.122
No (n = 56, 65.9%)	32.72 ± 26.23	
Sleep†		
Normal (n = 24, 28.3%)	39.16 ± 24.48*	0.001
Disturbed (n = 61, 71.8%)	16.79 ± 17.74*	
BMI**		
Normal (n = 56, 65.9%)	36.77 ± 27.39	0.716
Underweight (n = 16, 18.8%)	34.87 ± 13.50	
Overweight (n = 13, 15.3%)	28.02 ± 09.01	
Exercise†		
Insufficient (n = 77, 90.6%)	33.50 ± 31.05	0.418
Regular (n = 8, 9.4%)	39.75 ± 24.99	
Premorbid personality**	45.22 ± 23.90	
Peacefull (n = 35, 41.2%)		0.097
Aggresive (n = 23, 27.1%)	43.88 ± 26.29	
Withdrawn (n = 16, 18.8%)	43.00 ± 11.15	
Anxious (n = 11, 12.9%)	36.22 ± 10.19	

SD: Standard deviation; FIM: Functional independence measure; BT: Before treatment; AT: After treatment; BMI: Body mass index

* Significant difference (P < 0.050),

** Kruskal-Wallis test,

† Man-Whitney U test,

†† Wilcoxon signed rank test

Paolucci, et al. separated the patients who were similar age, disease severity, and rehabilitation condition into two groups and found that prognosis of women was poorer than that of men. They thought the cause of this depended on differences in the physiology between men and women; the dependency and insecurity of women performing in the rehabilitation were higher than these of men.^15^^,^^16^ In another study from China, men and women men and women for the motor recovery.

**Table 6:** The distribution and comparison of functional independence measure (FIM) levels in risk combination groups

**FIM score**	**BT (mean ± SD)**	**AT (mean ± SD)**	**BT-AT (mean ± SD)**
**Variable**			
2 risk factors combination (n = 30)	74.33 ± 34.03	118.13 ± 10.35	-37.80 ± 28.89
3 risk factors combination (n = 27)	88.25 ± 28.97	116.07 ± 16.10	-27.81 ± 20.09
4 risk factors combination (n = 5)*	45.06 ± 10.95	80.67 ± 11.50	-35.60 ± 22.45

The data analysis was done using Kruskal-Wallis test.

SD: Standard deviation; FIM: Functional independence measure; BT: Before treatment; AT: After treatment

* Significant difference (P < 0.050)

But pretreatment functional disability scores were over the age of 75 were compared in terms of prognosis, and found that the mortality rates were significantly higher in men at 12 months.^17^

In this study, no difference was found between more in women than in men; on the other hand, functional recovery scores were higher in women than in men. 

Although, we could not find any relationship among education and functional results in current study, it has been recorded that people with a low educational level and budget have less information about stroke; moreover their functional outcome is less, and also it has claimed that a low educational level may negatively affect the treatment compliance and medical suggesions.^10^^,^^18^ Besides, we could not find any association between marital status and functional outcome. However, Ng, et al. found that higher FIM scores were associated with single marital status.^19^

In a recent study, post-stroke obesity was a protective factor in men at 36 months, and the risk of mortality was decreased about 70%.^17^ However, in this study, motor and functional disabilities were more likely in overweight patients compared to those of normal weight.

For prediction of the stroke prognosis, accompanying medical conditions especially HT, DM, and cardiovascular diseases have been investigated. Although HT is one of the etiological factors of the stroke, there are conflicting results about the effect of blood pressure changes on the prognosis. Similar contradictory results are present in DM. In a study, patients with stroke and DM, had higher mortality rates, and were less likely to show clinical improvement.^18^

In another study, diabetes mellitus and HT did not affect total FIM scores.^19^ DM and increased number of co-morbidities were found to be related to prolonged hospital stay in previous studies done by Spratt, et al.^20^ and Lee, et al.^21^ Similarly, in the current study, pretreatment and posttreatment FIM levels were significantly lower in whom have 4 and more risk factors than those with 2 and more, also 3 and more risk factors.

In many studies, it has been shown that the maximum rate of oxygen consumption (VO_2_ max) significantly decreased after the stroke.^22^^,^^23^ However, we could not find any study that was investigated the effect of premorbid physical fitness. In our study, we could not find the effect of premorbid physical condition on Brunnstrom and FIM values.

In previous studies, sleep disorders have been reported as a negative factor in the prognosis of stroke.^24^ In our study, appropriate with literature, we found that functional outcome was less in patients with the sleep disorder.

Lee, et al. found that the post-stroke mortality rates were significantly higher in men at 12 months. Consequently, they concluded that smoking and alcohol expenditure prevalence were higher in men.^21^ In this study, it was also detected that motor recovery was less in smokers.

There was less study on the effect of personality characteristics on the prognosis. Elmstahl, et al. found that extrovert personality related with improved ADL.^25^ In the current study, motor and functional improvements were less in patients with the anxious personality trait.

The main limitation of this study is relatively small sample size, because the study was representative of stroke within the MCA territory, to ensure the homogeneity. However, to our knowledge, no studies to date have investigated the effect of premorbid features on rehabilitation outcome, or pay little attention to its importance. On the other hand in this study, we focused on patients’ lifestyle factors on rehabilitation outcome and found that many premorbid factors may affect the prognosis of patients with stroke.

## Conclusion

In conclusion, the major functional disability predictor in patients with stroke was having 4 and more risk factors (advanced age, the excess number of comorbidity, smoking, sleep disorders, obesity, and anxious personality). Consequently, while performing patients' rehabilitation programs, patients' lifestyle and risk factors would be useful parameters to predict functional outcome. There are few studies on this topic, which suggest the need for further studies with long-term follow-up.

## Notes:

**How to cite this article:** Karaahmet OZ, Umay E, Gurcay E, Serçe A, Gundogdu I, Cakci A. The effect of premorbid features on post-stroke rehabilitation outcome. Iran J Neurol 2018; 17(1): 38-46.
